# Comprehensive Sampling Across Seasons and Habitats Revealed the Composition and Functional Attributes of Gut Microbiota Enterotypes in Subterranean Rodents

**DOI:** 10.1002/ece3.73620

**Published:** 2026-05-04

**Authors:** Shien Ren, Jingge Wang, Dongni Guo, Jin Li, Yifan Zhao, Chongxuan Han, Xiaoning Nan

**Affiliations:** ^1^ Key Laboratory of National Forestry and Grassland Administration on Management of Western Forest Bio‐Disaster, College of Forestry Northwest A&F University Yangling Shaanxi China; ^2^ College of Animal Science and Technology Ningxia University Yinchuan Ningxia China

**Keywords:** enterotypes, Gansu zokor, gut microbiota, pest control, subterranean rodent

## Abstract

Several rodent species are considered global pests owing to their broad dietary habits, with stable gut microbiota enterotypes playing a vital role in host digestion. This study examined the Gansu zokor, a subterranean pest rodent endemic to the Loess Plateau. By extensive sampling across seasons and habitats, we characterized the gut microbiota enterotypes and clarified the mechanisms underlying their adaptation to diverse dietary resources. The gut microbiota of the Gansu zokor was categorized into two distinct enterotypes (E1 and E2), whose distribution varied markedly by season and habitat. The *α* and *β* diversity differed notably between the two enterotypes. Although Bacillota (formerly Firmicutes) and Bacteroidota dominated both enterotypes, the Bacillota/Bacteroidota ratio was markedly higher in E2, and this higher ratio was correlated with greater host body weight and BMI. Probiotic biomarkers such as *Muribaculum* and *Alistipes* were enriched in E1, whereas E2 was enriched in the *Lachnospiraceae NK4A136 group*, *Butyribacter*, and *Roseburia*. Potential pathogens such as *Bordetella*, *Acinetobacter*, and *Desulfovibrio* also serve as key biomarkers. E1 was enriched with genes involved in lipid metabolism, transport, and catabolism, while E2 was characterized by genes related to terpenoid and polyketide metabolism. Co‐occurrence networks revealed more interconnected interactions in E1, but higher modularity in E2. Stochastic processes (e.g., drift) governed microbial assembly in E1, whereas deterministic processes (e.g., homogeneous selection) dominated in E2. Enterotype stratification was closely correlated with host body size parameters and microbial *α* diversity. Research identifies two distinct enterotypes of zokors, clarifying their traits and links to food resource utilization. These findings illuminate the ecological adaptations of subterranean rodents and offer a theoretical basis for developing microbe‐based pest control strategies.

## Graphical abstract

The gut microbiota of Gansu zokor is classified into two enterotypes, which exhibit distinct seasonal and habitat distributions, microbial diversity, and community compositions dominated by different Bacillota‐to‐Bacteroidota ratios. These enterotypes correlate with host body size and are characterized by unique probiotic and pathogenic biomarkers.

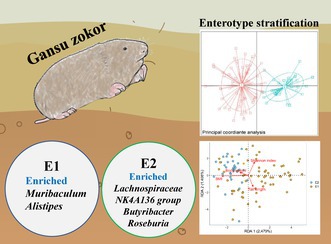

## 1 Introduction

Several rodents are global pests due to their broad ecological adaptability, with their diverse dietary habits serving as the foundation for thriving in varied environments. They consume most of the available plant species within their habitats (Lopes et al. 2020), and feed on parts such as fruits, seeds, leaves, roots, and stems, as well as mosses, lichens, and invertebrates (Butet and Delettre 2011). Recent studies showed that the gut microbiota plays a crucial role in the digestion and breakdown of dietary components (Zhu and Xu 2025). Herbivorous rodents such as the Alashan ground squirrel, Brandt's vole, and zokors harbor large amounts of cellulose‐degrading bacteria in their gut, enhancing their ability to digest plant cell walls (Chu et al. 2025; Li et al. 2024; Zou et al. 2025). In contrast, southern grasshopper mice, which consume a high proportion of insects, possess a gut microbiota enriched with chitinase genes, facilitating the breakdown of insect exoskeletons (Chu et al. 2025; Kohl et al. 2022). In herbivorous pests specifically, diets high in cellulose, hemicellulose, and polysaccharides cannot be digested by the host. Instead, these compounds are converted into short‐chain fatty acids (SCFAs) by carbohydrate‐active enzymes produced by the gut microbiota; these SCFAs are then utilized by the host as energy sources (Flint et al. 2012). Furthermore, the gut microbiota helps the host detoxify the toxic secondary metabolites present in the diet, thereby expanding the host's dietary niche (Kohl et al. 2014; Ren et al. 2021). Thus, the gut microbiota is essential for the ecological adaptability of pest species.

The gut microbiota is susceptible to regulation by various external environmental factors. Studies found that the gut microbiota of wild mice, North American red squirrels, and plateau pikas exhibited significant seasonal fluctuations (Maurice et al. 2015; Ren et al. 2017; Ren et al. 2024). Cross‐species comparative research revealed that habitat environments drove convergence in the gut microbiota of rodents (Lavrinienko et al. 2021; Wu, Zhou, Gu, et al. 2024). Despite its plasticity, the community structure of the gut microbiota does not exhibit a continuous gradient distribution but clusters into several distinct and relatively stable states. This discrete clustering pattern is defined as the “enterotype” (Arumugam et al. 2011; Lai et al. 2023). Enterotypes are typically classified based on their dominant bacterial genera. For example, a *Bacteroides*‐dominated enterotype specializes in degrading proteins and fats, whereas a *Prevotella*‐dominated enterotype is more adapted to a high‐carbohydrate diet (Wu et al. 2011). The concept of enterotypes provides new insights for the targeted modulation of gut microbiota for pest control (Zhang et al. 2023). Specifically, interference with core bacterial groups essential for host health or niche adaptability could impair host energy metabolism and stress resistance. This increased susceptibility to environmental pressures or natural enemies may ultimately suppress population growth and spread (Basit et al. 2025; Chen et al. 2020; Haider et al. 2025).

The Gansu zokor (*Eospalax cansus*) is a typical subterranean rodent native to the Loess Plateau and inhabits diverse environments, such as woodlands, farmlands, and grasslands. Zokors primarily feed on plant roots and rhizomes and have a broad dietary spectrum that encompasses nearly all the plant species in their habitats (Zhang et al. 2022). They cause considerable damage to major afforestation tree species, with reported damage rates of 43.5% for Mongolian pine, 55.2% for Chinese pine, and 10.1% for spruce (Han et al. 2009), posing a significant challenge to the implementation of the Grain for Green Project in Northwest China. In addition, their burrowing activities modify microtopography and exacerbate soil erosion (Chen et al. 2021). As a species that has undergone long‐term adaptation to extreme subterranean environments, the Gansu zokor may rely on its gut microbiota to assist in coping with hypoxic conditions and nutrient‐poor food sources, resulting in distinctive structural and functional microbial traits (Yang et al. 2025). Consequently, investigating the enterotypes of zokors not only elucidates the core microbial assemblages involved in host–environment interactions and the mechanisms underlying their adaptation to subterranean life but also provides a theoretical foundation for the development of novel, microbe‐based, eco‐friendly control strategies.

In this study, the gut microbiota of zokors was systematically investigated through comprehensive sampling across three seasons (spring, summer, and autumn) and three habitat types (woodlands, farmlands, and grasslands). By integrating body size parameters with 16S rRNA sequencing technology, we aimed to: (1) define the enterotypes of the zokor gut microbiota; (2) compare the microbial composition and metabolic potential between enterotypes, and identify dominant bacterial genera and interactions among core taxa; (3) analyze the assembly processes within enterotypes to reveal the ecological mechanisms driving microbial community formation; and (4) elucidate the associations between enterotypes, zokor body size, and microbial diversity. The identification and analysis of enterotypes in Gansu zokor not only advance our understanding of the ecological adaptability of subterranean rodents but also provide a foundation for the development of green control strategies based on gut microbial intervention. This research holds significant value for ecological conservation and the sustainable development of forestry.

## 2 Materials and Methods

### 2.1 Sample Collection

A total of 97 adult Gansu zokors were collected during spring (April), summer (July), and autumn (November) in the Ningxia Hui Autonomous Region. The sampling sites were located in Haiyuan County (36.28° N, 105.61° E; *n* = 43), Jingyuan County (35.55° N, 106.34° E; *n* = 17), and Xiji County (36.07° N, 105.78° E; *n* = 37) (Tables S1 and S2). Following capture, basic biological parameters, including body weight, body length, and tail length, were measured. Body mass index (BMI) was calculated as follows: BMI = body weight/(body length)^2^. Cecal content samples were then collected post euthanasia. To prevent cross‐contamination, all surgical instruments were sterilized with 75% ethanol prior to use. The collected cecal samples were aliquoted into 2 mL cryovials, flash‐frozen in liquid nitrogen, and transported to the laboratory for storage at −80°C. All experimental protocols received approval from the Ethics Committee of Northwest A&F University and were conducted in compliance with institutional animal welfare guidelines.

### 2.2 DNA Extraction and Sequencing

Total genomic DNA was isolated from gut content samples with the E.Z.N.A. Stool DNA Kit (Omega Biotek, USA) following the manufacturer's instructions. The integrity and concentration of the extracted DNA were assessed through 1.0% agarose gel electrophoresis and measurement with a NanoDrop 2000 spectrophotometer (Thermo Fisher Scientific, USA). Amplification of the V3–V4 hypervariable regions of the bacterial 16S rRNA gene was performed using the universal primers 341F (5′‐CCTAYGGGRBGCASCAG‐3′) and 806R (5′‐GGACTACNNGGGTATCTAAT‐3′). Polymerase chain reaction (PCR) was conducted in 20 μL reactions containing 4 μL of 5× Fast Pfu buffer, 2 μL of 2.5 mM dNTPs, 0.8 μL of each primer at a concentration of 5 μM, 0.4 μL of Fast Pfu polymerase, 10 ng of template DNA, and nuclease‐free water to volume. The thermal cycling protocol involved initial denaturation at 95°C for 5 min; 30 cycles of denaturation at 95°C for 30 s, annealing at 58°C for 30 s, and extension at 72°C for 45 s; and a final extension at 72°C for 10 min. PCR amplicons were visualized on a 2% agarose gel and purified using the AxyPrep DNA Gel Extraction Kit (Axygen Biosciences, USA). After quantification with a Qubit 3.0 fluorometer (Invitrogen, USA), the barcoded amplicons were pooled in equimolar amounts. The pooled DNA library was fragmented by ultrasonication and prepared using the NEBNext Ultra DNA Library Prep Kit (New England Biolabs, USA). Finally, paired‐end (PE300) sequencing was performed on an Illumina MiSeq platform according to the manufacturer's recommendations.

### 2.3 Bioinformatics Analysis

The raw paired‐end reads obtained from Illumina sequencing were initially demultiplexed to assign sequences to their respective samples. Quality control and filtering were applied based on Phred quality scores to retain high‐quality sequences. Overlapping regions between paired‐end reads were merged to reconstruct the full‐length amplicon sequences. Denoising was performed using the DADA2 algorithm (Callahan et al. 2016) within the QIIME2 environment to generate amplicon sequence variants (ASVs) and their corresponding abundance profiles. Taxonomic classification of ASVs was accomplished by aligning representative sequences against the SILVA v138.2 database (Quast et al. 2013) with the UCLUST algorithm (Edgar 2010). Finally, functional potential was assessed through PICRUSt2 (Douglas et al. 2020), with the predicted genes subsequently annotated according to the Kyoto Encyclopedia of Genes and Genomes (KEGG) database.

### 2.4 Machine Learning Model Screening

Six classical machine learning algorithms—*K*‐nearest neighbor, random forest, Gaussian naive Bayes, logistic regression, decision tree, and eXtreme gradient boosting—were employed to classify the samples and identify key biomarkers for the enterotype classification of zokors. The gut microbiota profiles were randomly divided into a training set (70%) for model development and a test set (30%) for performance evaluation. Model performance was evaluated using receiver operating characteristic (ROC) curves and the area under the curve (AUC). An ROC curve positioned closer to the top‐left corner indicates higher sensitivity and specificity, reflecting a strong ability to identify true positives and a low misclassification rate. The AUC serves as a quantitative measure, where values approaching 1 denote better discriminatory power of the model, suggesting that the identified biomarkers are effective for classification. All data preprocessing and modeling analyses were conducted in the R v4.3.1 statistical computing environment.

### 2.5 Ecological Network Construction

Co‐occurrence network analysis was performed to elucidate potential interactions among the gut microbiota of zokors. To enhance data quality, raw ASVs were filtered to remove low‐abundance (average relative abundance < 0.01%) and rare ASVs (occurrence frequency < 30% across all samples), thereby minimizing noise. Spearman rank correlation coefficients between pairwise ASVs were then computed employing the “psych” package (Yuen et al. 2018), with *p* values adjusted for multiple comparisons via the false discovery rate (FDR) approach (Benjamini et al. 2006). Significant correlations (Spearman's |*R*| > 0.6 and FDR‐corrected *p* < 0.05) were retained for network construction. This threshold is typically used in microbial network studies to capture robust and biologically meaningful associations while reducing the inclusion of weak or potentially spurious correlations (Fan et al. 2022). The microbial co‐occurrence network was visualized with Gephi v0.9.2 software (Jacomy et al. 2014), where nodes represent ASVs and edges denote significant correlations. To systematically characterize the network, topological properties were calculated at both the node level (degree, closeness centrality, betweenness centrality, and eccentricity) and network level (total nodes, total links, positive/negative links, average degree, density, average clustering coefficient, and average path length) for distinct gut enterotypes of zokors.

### 2.6 Microbial Community Assembly Processes

To investigate the formation mechanisms of distinct gut microbiota enterotypes in zokors, we assessed the relative influences of stochastic and deterministic processes. The neutral community model (NCM) was applied to identify the role of stochasticity in community assembly by examining the correlation between microbial occurrence frequency and relative abundance (Sloan et al. 2006). This model, which is grounded in the neutral theory that emphasizes random birth, death, and dispersal as key drivers of community structure, is widely used in microbial ecology (Hubbell 2001). The NCM was implemented using the “Hmisc” package in R v4.3.1, which fits the observed distribution of taxon abundance and frequency to a *β* distribution derived from neutral theory, thereby quantifying the extent to which stochastic processes influence community assembly.

To assess the comparative influence of deterministic and stochastic mechanisms on microbial community assembly, we utilized the “iCAMP” R package to compute the *β*‐nearest taxon index (*β*NTI) within a null model framework. Prior to analysis, ASVs with an average relative abundance lower than 0.01% were filtered out to minimize noise. The *β*NTI measures the normalized effect size of the *β*‐mean nearest taxon distance (*β*MNTD), incorporating phylogenetic information. A |*β*NTI| value ≥ 2 signifies that the observed phylogenetic turnover significantly exceeds what would be expected by random chance, indicating the predominance of deterministic processes. Conversely, a |*β*NTI| value < 2 suggests that phylogenetic turnover is not significant, suggesting that stochastic processes are the primary drivers (Stegen et al. 2012). To further elucidate the assembly mechanisms, the *β*NTI was combined with the Raup–Crick index calculated from Bray–Curtis distance (RCBray). This combination allowed us to categorize the assembly processes into five distinct ecological modes: homogeneous selection (*β*NTI < −2), heterogeneous selection (*β*NTI > 2), dispersal limitation (|*β*NTI| < 2 and RCBray > 0.95), homogenizing dispersal (|*β*NTI| < 2 and RCBray < −0.95), and drift or undominated processes (|*β*NTI| < 2 and |RCBray| < 0.95) (Stegen et al. 2015).

### 2.7 Niche Breadth Calculation

To classify microbial taxa according to their niche breadth, a permutation test was implemented using the “EcolUtils” package in R. This procedure randomized the occurrence frequencies of ASVs across samples for 1000 iterations, generating a null distribution of occurrence frequencies for each taxon. The 95% confidence intervals for the occurrence frequency of each species were then derived from this null distribution. In accordance with ecological theory, which posits that generalist species occupy broader fundamental niches than specialist species (Wilson and Hayek 2015), the taxa were categorized as follows: those with observed occurrence frequencies greater than the upper limit of the 95% confidence interval were identified as generalists; those with frequencies lower than the lower limit were classified as specialists; and the remaining taxa were designated as neutral species.

### 2.8 Statistical Analysis

To characterize the clustering patterns of gut microbiota enterotypes, we employed the partitioning around medoids (PAM) algorithm from the “cluster” package in R v4.3.1. The optimal number of clusters was identified by maximizing the Calinski–Harabasz (CH) index, calculated with the “clusterSim” package (Arumugam et al. 2011). UpSet plots were constructed using the “UpSetR” package to depict intersections of ASVs between groups (Conway et al. 2017). *β*‐diversity was assessed through principal coordinate analysis (PCoA) employing the Bray–Curtis distance matrix, implemented via the “vegan” package (Oksanen 2015). Permutational multivariate analysis of variance (PERMANOVA) was applied to quantify the contribution and statistical significance of treatment factors to the observed community variation. To exclude the influence of community dispersion on the PERMANOVA results, permutational analysis of multivariate dispersions (PERMDISP) was further employed to assess the homogeneity of dispersion between groups. Differential species between groups were determined by applying linear discriminant analysis effect size (LEfSe), employing a linear discriminant analysis (LDA) score threshold exceeding 2.5 and a statistical significance threshold of *p* < 0.05. The contribution of bacterial genera to differences in community composition across enterotypes was assessed using similarity percentage analysis (SIMPER). Microbial functional prediction data analysis was completed using STAMP v2.1.3 software (Parks et al. 2014). Redundancy analysis (RDA), performed with the “vegan” package (Oksanen 2015), was used to explore the associations between enterotype communities, body size parameters, and microbial *α* diversity indices. For comparisons between groups, either Student's *t*‐test or the Mann–Whitney *U* test was utilized, with *p* values being corrected for multiple comparisons through the FDR method (Benjamini et al. 2006).

## 3 Results

### 3.1 Identification of Enterotypes and Comparison of the Morphological Parameters of Zokors

**FIGURE 1:**
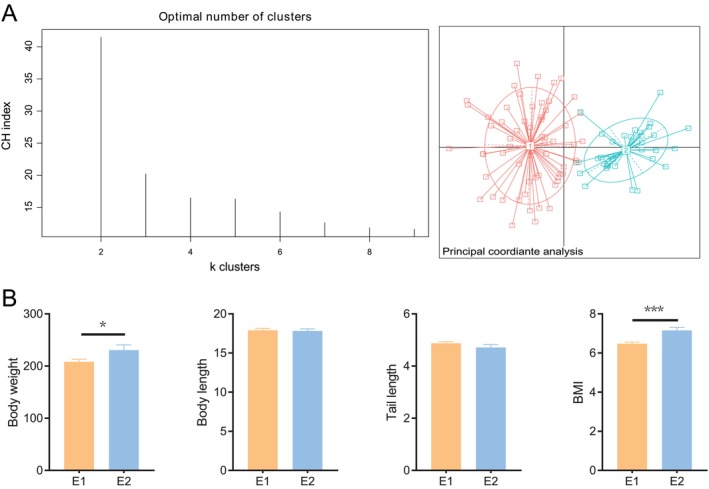
Identification of enterotypes and body size parameter differences in zokors. (A) Calinski–Harabasz (CH) index for enterotypes and principal coordinate analysis (PCoA) based on Jensen–Shannon distance. (B) Student's *t*‐test was used to examine differences in body size parameters (body weight, body length, tail length, and BMI) between enterotypes. *p < 0.05, ***p < 0.001.

On the basis of the results of the genus‐level Jensen–Shannon distance analysis, combined with the results of the CH index and silhouette score, the gut microbiota of 97 Gansu zokors were classified into two clusters, as validated by the optimal CH value; PCoA further revealed a clear separation in gut microbial community structure between the two clusters (Figure 1A). Accordingly, all the samples were defined as two enterotypes: enterotype 1 (E1, *n* = 63) and enterotype 2 (E2, *n* = 34). The seasonal distribution of enterotypes revealed that in spring, there were 37 of E1 and 31 of E2; in summer, there were 11 of E1 and 3 of E2; and in autumn, all were E1 (*n* = 15). The habitat distribution of enterotypes showed that in woodland, 22 were E1 and 18 were E2; in farmland, 25 were E1 and 12 were E2; and in grassland, 16 were E1 and 4 were E2 (Figure S1). Comparative analysis of morphological parameters indicated that body weight and BMI were significantly lower in the E1 group than in the E2 group (*p* < 0.05), while no significant differences were observed in body length or tail length between the two groups (Figure 1B).

### 3.2 Differences in Microbial Diversity and Composition Between Enterotypes

**FIGURE 2:**
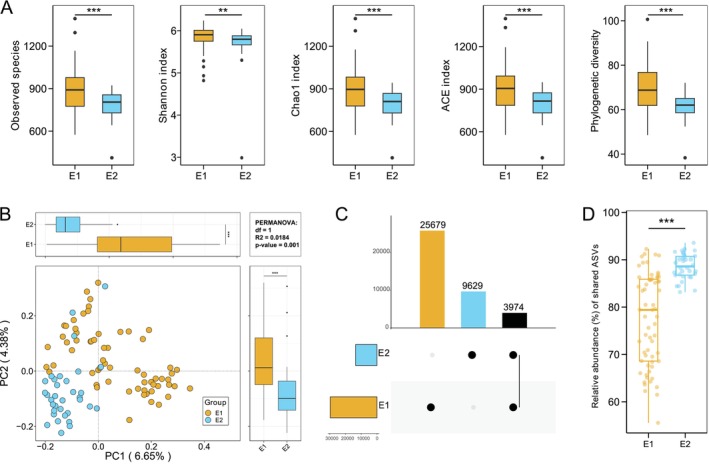
Microbial diversity of zokor enterotypes. (A) Alpha diversity (observed species, Shannon index, Chao1 index, ACE index, and phylogenetic diversity) differed significantly between the two enterotypes. (B) PCoA analysis based on Bray‐Curtis distance revealed distinct clustering of the two enterotypes, with clear separation along the PCo1 and PCo2 axes. (C) UpSet diagram displaying the number of ASVs shared between the two enterotypes. (D) Differences in the relative abundance of ASVs shared between the two enterotypes. Difference testing was performed using the Mann–Whitney *U* test. **p < 0.01, ***p < 0.001.

We further compared the microbial diversity between the two enterotypes at the ASV level. *α* diversity indices, including observed species, Shannon index, Chao1, ACE, and phylogenetic diversity, were all significantly higher in E1 than in E2 (Figure 2A). PCoA combined with PERMANOVA revealed a significant difference in gut microbial community structure between E1 and E2 (PERMANOVA: *R* ^2^ = 0.0184, *p* = 0.001), with clear separation along the PC1 and PC2 axes (*p* < 0.001) (Figure 2B). The PERMDISP test indicated no significant difference in multivariate dispersions between groups (*R* ^2^ = 0.538, *p* = 0.120), suggesting that the compositional difference detected by PERMANOVA was attributable to shifts in community centroid rather than heterogeneity in within‐group variability. UpSet diagram analysis showed that E1 contained 25,679 (65.37%) unique ASVs, E2 contained 9629 (24.51%) unique ASVs, and 3974 (10.12%) ASVs were shared between the two enterotypes (Figure 2C). Furthermore, the abundance of these shared ASVs was significantly lower in E1 than in E2 (Figure 2D).

**FIGURE 3:**
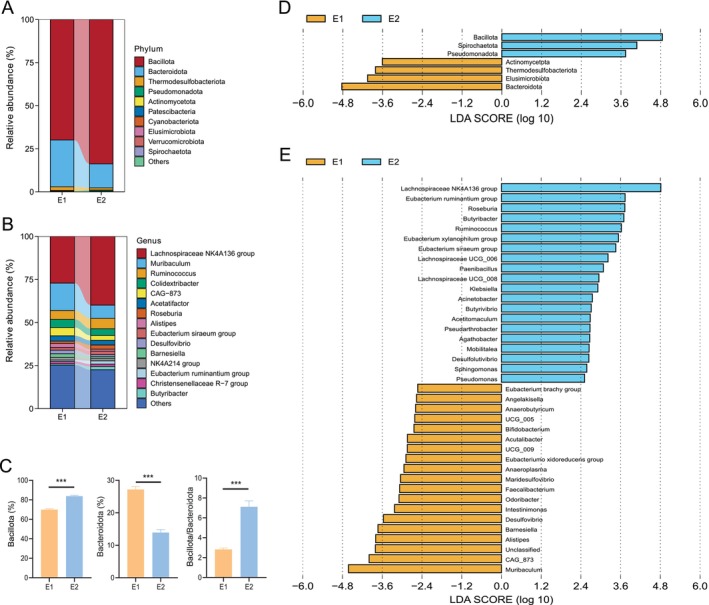
Taxonomic composition and differences of zokor enterotypes. (A, B) Major bacteria at the phylum and genus levels. (C) The Mann–Whitney *U* test was used to assess variations in major bacterial phyla between enterotypes. (D, E) LEfSe analysis identified bacteria with significant differences between enterotypes at the phylum and genus levels (LDA score > 2.5 and *p* < 0.05). ***p < 0.001.

At both the phylum and genus levels, the dominant taxa in the E1 and E2 groups were consistent. The predominant phyla were Bacillota (formerly Firmicutes) and Bacteroidota (Figure 3A), while the dominant genera included *Lachnospiraceae NK4A136 group*, *Muribaculum*, and *Ruminococcus* (Figure 3B). Comparative analysis revealed that the relative abundance of Bacillota was significantly lower in E1 than in E2, whereas Bacteroidota was significantly enriched in E1. Consequently, the Bacillota/Bacteroidota ratio was significantly higher in E2 than in E1 (Figure 3C). LEfSe analysis at the phylum level confirmed this distinction and further identified Actinomycetota, Thermodesulfobacteriota, and Elusimicrobiota as significantly enriched in E1, while Spirochaetota and Pseudomonadota were enriched in E2 (Figure 3D). At the genus level, LEfSe results showed that 19 genera, including *Muribaculum*, *CAG_873*, and *Alistipes*, were enriched in the E1 group, while 20 genera, such as *Lachnospiraceae NK4A136 group*, *Eubacterium ruminantium group*, and *Roseburia*, were enriched in the E2 group (Figure 3E).

**FIGURE 4:**
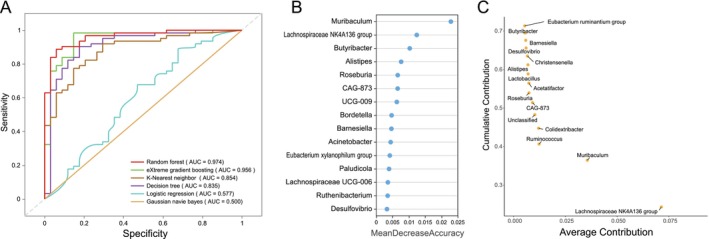
Identification of key bacterial genera crucial for zokor enterotype classification. (A) ROC curves were plotted at the genus level using six methods (random forest, eXtreme gradient boosting, *K*‐nearest neighbor, decision tree, logistic regression, and Gaussian naive Bayes). (B) Key bacterial genera identified by the random forest model for enterotype classification. The horizontal axis represents the contribution degree of bacteria to the model's predictive accuracy (a higher value indicates greater importance), and the vertical axis lists the top 15 important genera. (C) Key bacterial genera identified by SIMPER analysis for enterotype classification. The horizontal axis represents the average contribution of bacteria to intergroup differences, and the vertical axis represents the cumulative contribution of the top 15 genera.

To further identify biomarkers that significantly contribute to intergroup differences, we systematically evaluated the microbial data using six machine learning algorithms. The results indicated that the random forest model achieved the best performance across various evaluation metrics (AUC = 0.974) (Figure 4A). Based on this model, we identified a set of characteristic bacterial genera, including *Muribaculum*, *Lachnospiraceae NK4A136 group*, and *Butyribacter*, suggesting their potential as key microbial markers for distinguishing zokor gut types (Figure 4B). Further validation using SIMPER analysis revealed that the *Lachnospiraceae NK4A136 group*, *Muribaculum*, and *Ruminococcus* contributed substantially to intergroup differences, which was similar to the results obtained from the random forest analysis (Figure 4C).

### 3.3 Differences in Microbial Functions Between Enterotypes

**FIGURE 5:**
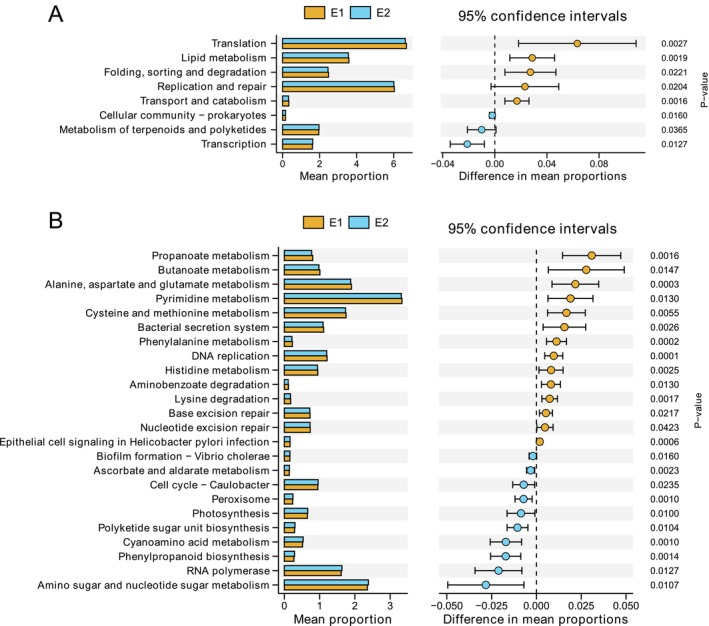
Functional category differences between enterotypes in zokors. The Mann–Whitney *U* test was used to analyze the differences in functional categories at (A) KEGG level 2 and (B) KEGG level 3 between enterotypes.

Microbial functional analysis revealed significant differences in overall metabolic potential between the E1 and E2 groups (PERMANOVA: *R* ^2^ = 0.1987, *p* = 0.001) (Figure S2). At the KEGG level 2 functional hierarchy, genes associated with translation, lipid metabolism, folding, sorting and degradation, and replication and repair were significantly enriched in the E1 group (*p* < 0.05). In contrast, genes related to transcription, metabolism of terpenoids and polyketides, and cellular community—prokaryotes were significantly enriched in the E2 group (*p* < 0.05) (Figure 5A). Further analysis at KEGG level 3 showed that 14 functional categories were significantly enriched in the E1 group, while 10 categories were significantly enriched in the E2 group (*p* < 0.05) (Figure 5B).

### 3.4 Microbial Interaction Network of Enterotypes

**FIGURE 6:**
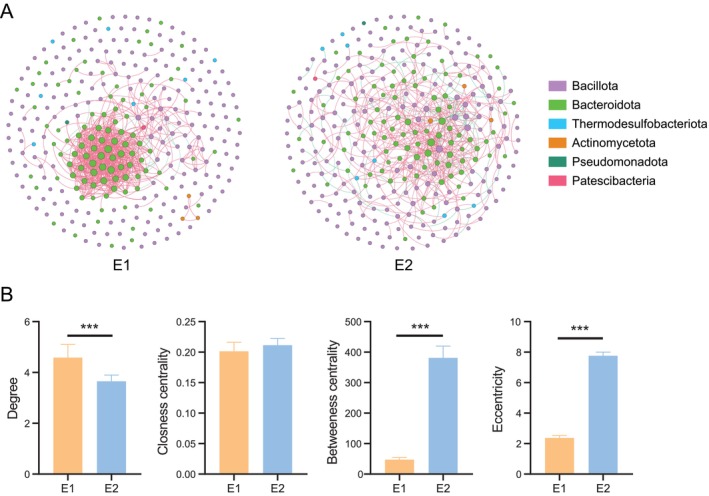
Microbial co‐occurrence network of zokor enterotypes. (A) The microbial co‐occurrence network of zokor enterotypes based on Spearman correlation. (B) Differences in node‐level topological features (degree, closeness centrality, betweenness centrality, and eccentricity) of the microbial co‐occurrence network between enterotypes were analyzed using the Mann–Whitney *U* test. ***p < 0.001.

Network analysis revealed distinct differences in microbial interaction patterns between the two enterotypes (Figure 6A). The E1 network comprised 310 nodes and 710 edges (97.0% positive interactions), whereas the E2 network consisted of 310 nodes and 566 edges (82.7% positive interactions). In terms of global topological properties, compared with the E2 network, the E1 network exhibited a higher average degree, density, and average clustering coefficient, but lower modularity and average path length (Table S3). At the node level, the degree was significantly elevated in the E1 group, whereas betweenness centrality and eccentricity were notably reduced. In contrast, closeness centrality revealed no significant variation between the E1 and E2 groups (Figure 6B).

### 3.5 Microbial Community Assembly Processes and the Niche Breadth of Enterotypes

**FIGURE 7:**
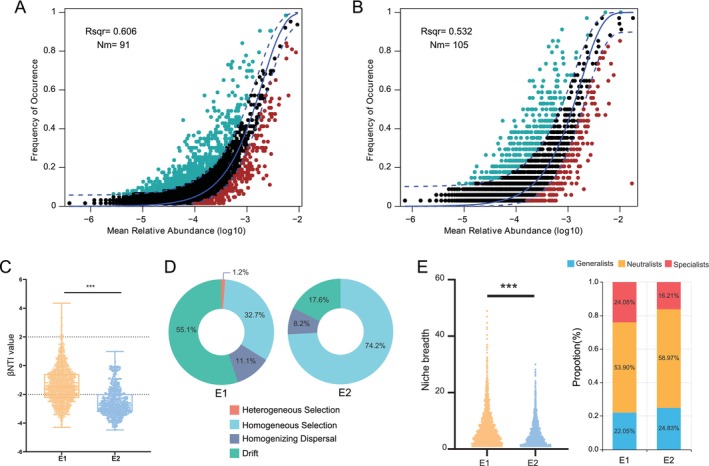
The assembly process and niche breadth of zokor enterotype communities. (A) Fit of the neutral community model (NCM) in the (A) E1 and (B) E2 communities. Solid lines illustrate the fit of the neutral model, with the upper and lower dashed lines denoting the 95% confidence intervals predicted by the model. ASVs that appear more or less frequently than the NCM predictions are displayed in distinct colors. Nm corresponds to the product of metacommunity size and immigration, and *R* ^2^ reflects the goodness‐of‐fit of the model. (C) BetaNTI values calculated for different community types. (D) Relative contributions of ecological processes in the assembly of the two enterotype communities. (E) Niche breadth of the two enterotype communities and the relative contributions of generalists and specialists. Statistical analyses were conducted utilizing the Mann–Whitney *U* test. ***p < 0.001.

NCM was utilized to investigate the possible influence of stochastic mechanisms on the formation of the two enterotype microbial communities. The findings displayed that the neutral community model effectively captured the relationship between the frequency of ASV occurrence and variations in their proportional abundance. Specifically, in the E1 group, the model explained 60.6% of the community variation, with an Nm value of 91 (Figure 7A); whereas in the E2 group, it accounted for 53.2% of the community variation, with an Nm value of 105 (Figure 7B).

Null model analysis revealed that the *β*NTI values in the E1 group were markedly greater than those in the E2 group (*p* < 0.001) (Figure 7C). Niche‐based process analysis indicated that the assembly of microbial communities in both enterotypes was primarily driven by homogeneous selection, homogenizing dispersal, and drift. Notably, the proportion of homogeneous selection in E1 (32.7%) was lower than that in E2 (74.2%), suggesting that community assembly in E2 was more strongly influenced by convergent environmental filtering. In contrast, the proportion of homogenizing dispersal was greater in E1 (11.1%) than in E2 (8.2%), reflecting a stronger dispersal capability in E1. Furthermore, the proportion of drift was significantly greater in E1 (55.1%) than in E2 (17.6%), indicating that stochastic processes contributed more importantly to the assembly of E1 communities (Figure 7D).

Niche breadth analysis indicated that the niche width of the E1 community was significantly greater than that of the E2 community (*p* < 0.001), suggesting that the E1 community overall possesses a broader resource utilization capacity. Community composition analysis revealed that the proportion of generalists in E1 (22.05%) was lower than that in E2 (24.83%), indicating higher resource heterogeneity in the E1 habitat, which favors the differentiation and coexistence of specialists. These results are also consistent with the higher proportion of drift processes observed in the E1 community. On the other hand, the proportion of specialists in E1 (24.05%) was higher than that in E2 (16.21%), reflecting stronger environmental filtering in the E2 habitat, which promoted the dominance of generalists because of their broad resource utilization capabilities. This trend aligns with the dominant role of homogeneous selection processes in E2 (Figure 7E).

### 3.6 Association of Enterotype With Body Size Parameters and *α* Diversity

**FIGURE 8:**
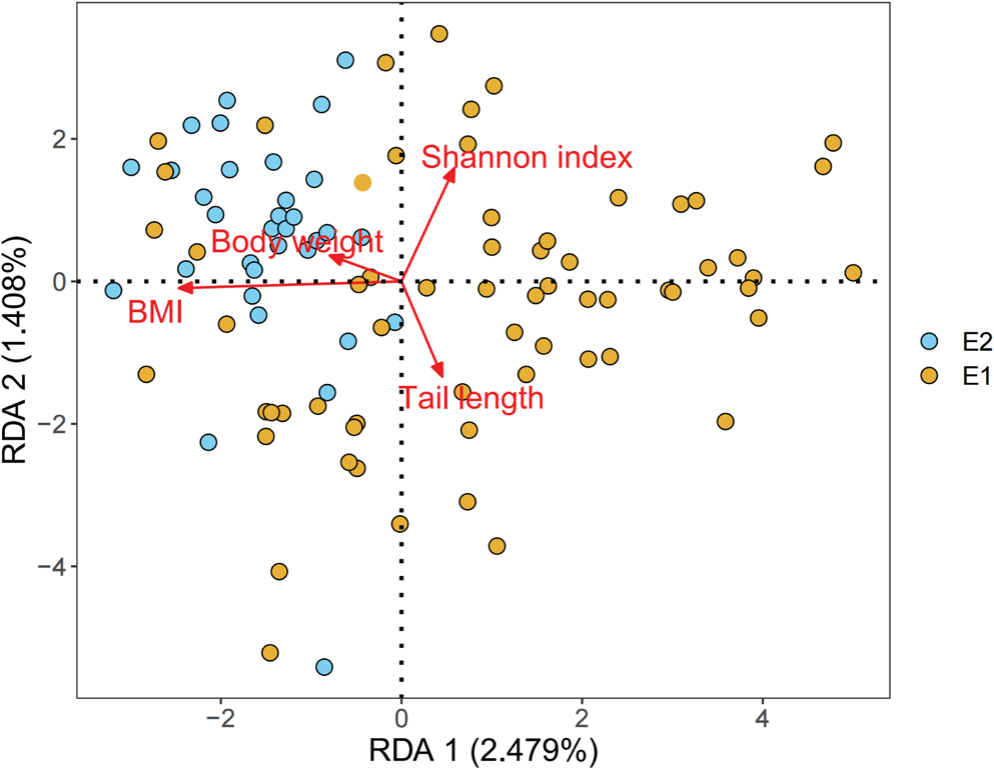
Redundancy analysis (RDA) of zokor enterotype communities with body size parameters and microbial alpha‐diversity. The direction and length of the arrows represent the relationships between the elements and the enterotype communities.

To mitigate the influence of multicollinearity on the results of redundancy analysis, Spearman correlation analysis was first conducted to evaluate the correlations among the various *α* diversity indices of the gut microbiota and among the different body size parameters (Tables S4 and S5). Subsequently, variables with an absolute correlation coefficient greater than 0.6 were excluded. Finally, the remaining variables, together with the enterotype data, were included in the redundancy analysis. Redundancy analysis revealed significant associations between the zokor's enterotypes and body size parameters as well as gut microbial *α* diversity (*F* = 1.417, *p* = 0.001). Tail length, BMI, and Shannon index all showed significant correlations with enterotypes (*p* < 0.01) (Figure 8 and Table S6).

## 4 Discussion

Our multiseason and multihabitat sampling revealed that the gut microbiota of the subterranean pest rodent, the Gansu zokor, can be classified into two distinct enterotypes. Both enterotypes were predominantly composed of Bacillota and Bacteroidota; however, the Bacillota/Bacteroidota ratio was significantly higher in E2 than in E1. This elevated ratio correlated with greater host body weight and BMI in E2, which is consistent with the understanding that a higher Bacillota/Bacteroidota ratio enhances energy harvest in the host (Zhang et al. 2025). Cross‐species comparisons indicated that animals occupying similar environments tend to share convergent enterotypes (Liu et al. 2025). Our results suggest that enterotypes are influenced by both seasonal and habitat factors. Specifically, habitats modulate enterotypes by altering dietary nutrient composition through available plant species. For example, woodland vegetation—characterized by higher cellulose content—favored the E2 enterotype, which is marked by increased Bacillota abundance and supports the digestion of fibrous plants. In contrast, the E1 enterotype, enriched with Bacteroidota, aligned with diets richer in carbohydrates commonly found in farmland and grassland habitats. Seasonal shifts further directly impact food nutritional quality as vegetation transitions between the growth and senescence phases, thereby driving enterotype transitions. Thus, enterotype switching represents an adaptive strategy of the gut microbiota to optimize host utilization of dynamically fluctuating food resources (Guo et al. 2021).

Random forest analysis integrated with species contribution analysis identified key bacterial genera driving enterotype divergence. The genera *Muribaculum* and *Alistipes*, belonging to Bacteroidota, were significantly enriched in E1. These genera are characterized as polysaccharide metabolism specialists and encode a wide array of carbohydrate‐active enzymes (Facimoto et al. 2025; Yamane et al. 2021). They exhibit anti‐inflammatory potential and likely contribute to intestinal barrier function and immunoregulatory balance (Lin et al. 2025; Wang, Wang, et al. 2025; Wang, Kang, et al. 2025). In E2, the *Lachnospiraceae NK4A136 group*, *Butyribacter*, and *Roseburia*, affiliated with Bacillota, were prominently enriched. These taxa are adept at fermenting dietary fibers to produce beneficial metabolites such as butyrate and propionate (Biddle et al. 2013; La Rosa et al. 2019; Zou et al. 2021). Their abundance is positively correlated with gut microbiota stability and anti‐inflammatory effects, demonstrating protective effects in various disease models (Ma et al. 2020; Tamanai‐Shacoori et al. 2017). Similarly, bacteria involved in food fermentation serve as key indicators of enterotypes in striped field mice (Wu, Zhou, Yang, et al. 2024). Notably, potential pathogens within Proteobacteria, including *Bordetella* (Mohamed et al. 2023), *Acinetobacter* (Harding et al. 2018), and *Desulfovibrio* (Singh et al. 2023), were identified as critical enterotype biomarkers. These findings imply that the suppression of specific food‐degrading beneficial bacteria in the zokor gut may facilitate pathogenic bacterial proliferation, suggesting a potential strategy for rodent pest control.

Functional profile analysis revealed distinct metabolic potentials between the two enterotypes. E1 is primarily distributed in farmland and grassland habitats, where diets may contain relatively high levels of crude fat. This may explain the enrichment of genes related to lipid metabolism, transport, and catabolism in the E1 gut microbiota. Enhanced lipid metabolic capacity can improve the efficiency of energy extraction, storage, and membrane biosynthesis, thereby supporting host energy balance in environments where fat‐rich resources are relatively readily available (Tracey et al. 2018). In contrast, woodland habitats are dominated by Pinaceae plants, such as Mongolian pine and Chinese pine, which are rich in terpenoids and polyketides—classes of plant secondary metabolites known for their antimicrobial and defensive properties (Rogachev and Salakhutdinov 2015; Yu et al. 2025). Similarly, E2 exhibited a higher abundance of genes associated with terpenoid and polyketide metabolism, suggesting a potential adaptive response of the gut microbiota to these chemically complex diets. The gut microbiota may protect the host against xenobiotics by degrading or biotransforming these compounds into less harmful forms or by facilitating their excretion (Gruszecka‐Kosowska et al. 2022). Furthermore, metabolic intermediates generated during terpenoid degradation may serve as alternative carbon and energy sources, thereby enhancing nutrient acquisition in resource‐limited woodland environments (Ghitti et al. 2024). Importantly, microbial communities are known to exhibit functional redundancy and plasticity, wherein small but consistent changes across multiple pathways can lead to ecologically significant outcomes (Louca et al. 2018; Liang et al. 2024). Therefore, although the observed differences in pathway abundance are relatively modest, their consistency across functionally related metabolic pathways supports the notion of adaptive fine‐tuning of the gut microbiome in response to habitat‐specific dietary pressures.

Network analysis identified unique patterns of microbial interactions distinguishing the E1 and E2 enterotypes. The microbial community in E1 exhibited a higher number of connections, whereas the network in E2 showed greater modularity. The broad niche breadth and high proportion of specialized species in E1 suggest significant environmental heterogeneity, potentially driven by diverse or spatiotemporally variable resources, which promotes the differentiation and coexistence of specialized taxa. This pattern aligns with an assembly mechanism dominated by stochastic processes (e.g., drift). In contrast, the narrower niche breadth and prevalence of generalist species in E2 indicate stronger environmental filtering, where generalists dominate via broad‐spectrum resource utilization. This is consistent with the greater influence of homogeneous selection on ecological processes.

Recent studies revealed that enterotypes are regulated by multiple factors across species. Human enterotypes are primarily associated with dietary factors (Liang et al. 2017; Wu et al. 2011; Zhong et al. 2019), whereas the enterotypes of plateau pikas are influenced by both diet and altitude (Fan et al. 2020; Yu et al. 2022). Similarly, African buffalo are significantly correlated with diet and enterotype at the population level (Couch et al. 2021), and altitude and body condition impact enterotype variation in striped field mice (Wu, Zhou, Yang, et al. 2024). Our research further indicated that enterotypes are not only modulated by body size parameters but also closely linked to the community structure, functional modules, and metabolic characteristics of the gut microbiota. Notably, enterotypes have recently been explored as monitoring indicators for the reintroduction of captive animals into the wild (Huang et al. 2024). Given that enterotypes profoundly affect food digestion efficiency, nutrient metabolism pathways, immune regulation capacity, and tolerance to specific toxic substances in rodents, we can conceptualize pest rodent populations as assemblages of functionally distinct enterotype subpopulations rather than homogeneous groups. This perspective offers new insights for targeted control strategies. Intervention strategies could involve the precise regulation of core microbiota functions within specific enterotypes. For example, designing phages or antimicrobial peptides to target and inhibit these core functions could compromise the nutritional metabolism of individuals with that enterotype, thereby reducing their fitness. On the other hand, long‐term monitoring of enterotype proportions in wild populations can help predict outbreak risks. For example, a significant increase in the proportion of a high‐energy acquisition enterotype often indicates sufficient food resources in the environment, suggesting that the population may be entering a rapid expansion phase. Thus, control measures should be deployed to prevent large‐scale damage. In brief, as an integrative indicator that incorporates host physiology, microbial function, and environmental factors, enterotypes hold significant potential for research on species adaptation, population dynamics monitoring, and precise ecological intervention.

This study has the following limitations. First, the sampling was restricted to specific regions within the range of the Gansu zokor, and the collection efforts across different locations, habitat types, and seasons were not equally distributed. While this reflects the practical challenges of field sampling in subterranean rodents, it may limit the generalizability of the observed enterotype–habitat associations. Second, this study relied on 16S rRNA gene amplicon sequencing, which provides predictive functional profiles via PICRUSt2. Future research should incorporate metagenomic or metatranscriptomic approaches to validate the functional differences identified here. Finally, the observed relationships between the gut microbiota composition and host phenotypic traits are inherently correlated, and experimental methods such as dietary manipulation or microbiota transplantation are needed to establish causal relationships.

## 5 Conclusions

In summary, through comprehensive sampling across seasons and habitats, we elucidated that the gut microbiota of Gansu zokor can be classified into two distinct enterotypes. Further investigations revealed that these two enterotypes are associated with divergent taxonomic compositions and metabolic potentials. The assembly processes and niche breadths of the microbial communities were also distinctly different between the two enterotypes. Association analyses identified significant correlations between the enterotypes and host morphometric traits as well as microbial *α* diversity. Future studies should employ larger sample sizes to investigate the potential existence of additional enterotypes in zokors and to experimentally validate the functional impact of enterotypes on host physiology. The results of the current study highlight the close relationship between enterotypes and foraging ecology in subterranean rodents, providing insights into ecological adaptation and a theoretical foundation for microbiome‐based pest management strategies.

## Author Contributions

**Shien Ren:** conceptualization (equal), data curation (equal), formal analysis (equal), funding acquisition (equal), investigation (equal), methodology (equal), validation (equal), visualization (equal), writing – original draft (equal). **Jingge Wang:** data curation (equal), formal analysis (equal), methodology (equal), validation (equal), visualization (equal). **Dongni Guo:** data curation (equal), formal analysis (equal), investigation (equal), software (equal). **Jin Li:** data curation (equal), formal analysis (equal), investigation (equal). **Yifan Zhao:** formal analysis (equal), investigation (equal). **Chongxuan Han:** writing – review and editing (equal). **Xiaoning Nan:** conceptualization (equal), funding acquisition (equal), investigation (equal), project administration (equal), resources (equal), supervision (equal), visualization (equal), writing – review and editing (equal).

## Funding

This work was supported by the National Forestry and Grassland Administration Project (202401‐10‐5) and the Chinese Universities Scientific Fund (2452024162).

## Ethics Statement

All experimental procedures were approved by the Ethics Committee of Northwest A&F University and adhered to institutional animal welfare regulations.

## Conflicts of Interest

The authors declare no conflicts of interest.

## Supporting information


**Table S1:** Overview sampling information for Gansu zokor.
**Table S2:** Specific sampling information for Gansu zokor.
**Table S3:** Network parameters of the two enterotypes.
**Table S4:** Spearman correlation among alpha diversity indices of the gut microbiota in Gansu zokor.
**Table S5:** Spearman correlation among body size parameters in the Gansu zokor.
**Table S6:** Table of RDA results.

**Figure S1:** Distribution of zokor enterotypes. (A) Seasonal and (B) habitat distribution of zokor enterotypes.
**Figure S2:** PCoA analysis of functional profiles based on Bray‐Curtis distance for the two enterotypes of zokors.

## Data Availability

All raw sequences in this study were submitted to the National Center for Biotechnology Information database with accession number PRJNA1430205.
