# Supplementary material for: Comprehensive Sampling Across Seasons and Habitats Revealed the Composition and Functional Attributes of Gut Microbiota Enterotypes in Subterranean Rodents
Source: Ecol Evol. 2026 May 4;16(5):e73620. doi: 10.1002/ece3.73620 (PMC13139636; doi:10.1002/ece3.73620)
Supplement: Supplementary file 2 — Figure S1: Distribution of zokor enterotypes. (A) Seasonal and (B) habitat distribution of zokor enterotypes. Figure S2: PCoA analysis of functional profiles based on Bray‐Curtis distance for the two enterotypes of zokors. [file ECE3-16-e73620-s001.docx]

**Supplementary Information for:**

**Comprehensive Sampling Across Seasons and Habitats Revealed the Composition and Functional Attributes of Gut Microbiota Enterotypes in Subterranean Rodents**


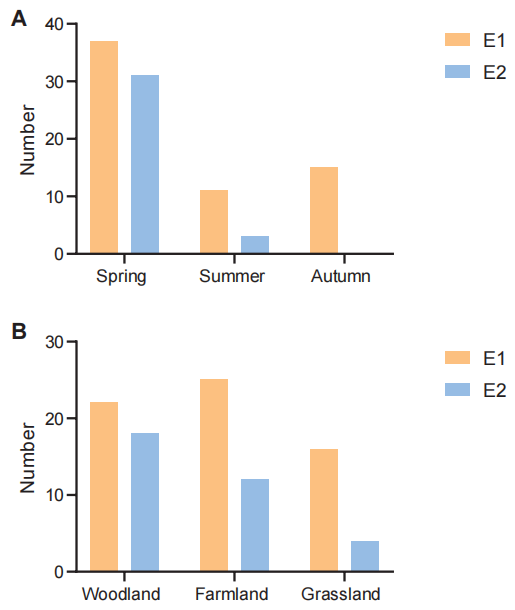


Figure S1 Distribution of zokor enterotypes. (A) Seasonal and (B) habitat distribution of zokor enterotypes.


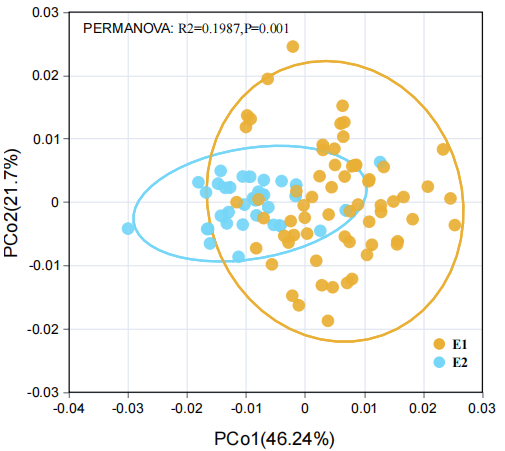


Figure S2 PCoA analysis of functional profiles based on Bray-Curtis distance for the two enterotypes of zokors.
